# Two RSV Platforms for G, F, or G+F Proteins VLPs

**DOI:** 10.3390/v12090906

**Published:** 2020-08-19

**Authors:** Binh Ha, Jie E. Yang, Xuemin Chen, Samadhan J. Jadhao, Elizabeth R. Wright, Larry J. Anderson

**Affiliations:** 1 Division of Pediatric Infectious Diseases, Emory University School of Medicine and Children’s Healthcare of Atlanta, Atlanta, GA 30322, USA; binh.ha@emory.edu (B.H.); xchen26@emory.edu (X.C.); samadhan.jadhao@emory.edu (S.J.J.); 2 Department of Biochemistry, University of Wisconsin, Madison, WI 53706, USA; jyang525@wisc.edu; 3 Cryo-Electron Microscopy Research Center, Department of Biochemistry, University of Wisconsin, Madison, WI 53706, USA; 4 Morgridge Institute for Research, Madison, WI 53715, USA

**Keywords:** respiratory syncytial virus (RSV), virus-like particles (VLPs), vaccine, electron microscopy (EM), mouse model

## Abstract

Respiratory syncytial virus (RSV) causes substantial lower respiratory tract disease in children and at-risk adults. Though there are no effective anti-viral drugs for acute disease or licensed vaccines for RSV, palivizumab prophylaxis is available for some high risk infants. To support anti-viral and vaccine development efforts, we developed an RSV virus-like particle (VLP) platform to explore the role RSV F and G protein interactions in disease pathogenesis. Since VLPs are immunogenic and a proven platform for licensed human vaccines, we also considered these VLPs as potential vaccine candidates. We developed two RSV VLP platforms, M+P and M+M2-1 that had F and G, F and a G peptide, or a truncated F and G on their surface. Immunoblots of sucrose gradient purified particles showed co-expression of M, G, and F with both VLP platforms. Electron microscopy imaging and immunogold labeling confirmed VLP-like structures with surface exposed projections consistent with F and G proteins. In mice, the VLPs induced both anti-F and -G protein antibodies and, on challenge, reduced lung viral titer and inflammation. These data show that these RSV VLP platforms provide a tool to study the structure of F and G and their interactions and flexible platforms to develop VLP vaccines in which all components contribute to RSV-specific immune responses.

## 1. Introduction

Respiratory Syncytial Virus (RSV) [1] was quickly recognized as a significant pediatric pathogen after its discovery in the 1950s. RSV causes upper and lower respiratory tract infections including bronchitis, bronchiolitis, and pneumonia. Most children are infected by two years of age [2] but then re-infections occur throughout life. The elderly and individuals with chronic cardiac or pulmonary disease, or immune-compromising conditions are at higher risk for severe complications with re-infection. It is estimated that globally, per year, there are more than 33 million cases of RSV-associated infections that result in 95,000–150,000 RSV deaths in children less-than five years of age, mostly in developing countries [3]. RSV–related deaths are rare in the United States. However, RSV infections are responsible for an estimated 60,000–170,000 hospitalizations each year in children less-than five years of age [4,5]. Additionally, infants hospitalized with RSV infection are prone to subsequent development of obstructive airway diseases and asthma [6,7]. The substantial global disease burden has made RSV a high priority for vaccine and anti-viral drug development. Currently, there are no effective anti-viral drugs for the acute infection or vaccines available [8]. Palivizumab prophylaxis is available for some high risk infants.

Understanding the biology and pathogenesis of RSV infection is important to development of vaccines and anti-viral drugs. RSV is a single-stranded, negative sense RNA virus belonging to genus *Orthopneumovirus* in the family *Pneumoviridae* with two distinct antigenic groups, A and B [9]. The RSV genome of approximately 15.2 kb includes 10 genes that encode for 11 proteins [10]. Three glycoproteins, fusion (F), attachment (G), and small hydrophobic (SH), are expressed on the virion envelope. The F and G proteins, however, are the only ones shown to induce effective neutralizing antibodies and longer-term protective immunity. The F protein is more conserved among RSV strains and induces cross-protective immunity and is the most effective at inducing neutralizing antibodies. The SH protein does induce some protection likely through Fc receptor mediated activity, such as antibody-dependent cellular cytotoxicity or complement activation [11]. Most neutralizing antibodies in human serum specimens are against the pre-fusion form of F [12]. In fact, pre-fusion F is currently a prime candidate for RSV subunit vaccines [13]. The G protein, though eliciting less potent neutralizing antibodies, has been shown to be an important factor for RSV disease pathogenesis, making it also a candidate for inclusion in an RSV vaccine [14]. Structurally, the G protein consists of a conserved region that contains a CX3C chemokine motif that enables its binding to the CX3C chemokine receptor, CX3CR1, which induces responses similar to those induced by the CX3C chemokine fractalkine [15]. In fact, our studies in the BALB/c mouse model indicate that G induces disease causing inflammatory responses that can be blocked by inhibiting G binding to CX3CR1 with passive administration of an anti-G monoclonal antibody [16,17,18], G peptide vaccine-induced antibodies [19,20,21], or by mutating the CX3C motif. G binding to CX3CR1 also mediates infection of primary human airway epithelial cells and CX3CR1 is considered an RSV receptor in these cells [22,23]. The functions of F and G in infection and disease pathogenesis continue to be important areas for investigation but their interaction has received little attention. F and G interactions are suggested by co-immunoprecipitation experiments, cryo-electron tomography (cryo-ET) studies of G’s effect on F in virions, and the need for both a mutated F and G to induce lung mucus in mice [16,24,25,26,27].

We chose to develop RSV platform based VLPs to support studies of F and G interactions and to develop candidate vaccines. An RSV platform VLP should mimic the natural structure of the F and G proteins better than other platforms and all components have the potential to contribute to vaccine-induced RSV immunity. RSV VLPs have been developed using the Newcastle disease, influenza, or bacteriophage P22 platforms and incorporated the RSV F and/or G proteins or M and M2 proteins [28,29,30]. RSV VLPs produced from an RSV platform have been described and were composed of F, M, nucleoprotein (N), and phosphoprotein (P) proteins; F, G, and M proteins; or F, M, and P proteins [31,32,33]. We describe successful development of RSV VLPs that incorporated F and/or G with the RSV M and P or M and M2-1. We developed VLPs with M and M2-1, in addition to those previously described with M and P, because M2-1 is thought to be important for structural stability of RSV [26]. We successfully generated RSV VLPs with surface expression of F and G, F and a G peptide, and F without most of its extracellular domain and G and demonstrated their immunogenicity and ability to protect RSV challenged mice.

## 2. Materials and Methods

### 2.1. Cells, Media, and Plasmids

All restriction enzymes were obtained from New England Biolabs (Ipswich, MA, USA). 293F cells were stably transfected with plasmid pcDNA6/TR and cultured in freestyle 293 media (Gibco, Thermo Fisher Scientific, Waltham, MA, USA) on a shaker at 37 °C, 8% CO_2_. pcDNA3.1 DNA plasmids containing codon-optimized RSV genes M, M2-1, P, G, and F from the A2 strain were from Dr. Martin Moore (now at Meissa Vaccines Inc, Redwood City, CA, USA). The plasmids were digested by KpnI and XhoI enzymes and cloned into KpnI and XhoI double digested pcDNA4/TO or pcDNA5/TO vector. Human codon-optimized truncated G consisting of amino acids 1-86+155-206 was synthesized by Genescript (Piscataway, NJ, USA). The gene provided in pUC57 plasmid was digested by BamHI and XhoI restriction enzymes and cloned into BamHI and XhoI digested pcDNA5/TO vector. All genes were sequence confirmed prior to transfection. To generate VLPs, 293F cells were sequentially and stably transfected with the RSV genes noted below. We chose stably instead of transiently transfected cells because they provide a more consistent source of VLPs with a known composition.

### 2.2. Virus-Like Particle Expression and Purification

A total of 20–30 × 10^6^ 293F cells were induced with 2 μg/mL of doxycycline for 72 h. Cells were centrifuged at 300× *g* for 10 min and the VLP-containing media was filtered through 0.45 μm filter followed by centrifugation through 20% sucrose cushion at 12,200× *g* for 2 h at 4 °C (SW Ti 32 rotor, Optima L-90K Ultracentrifuge, Beckman Coulter, (Indianapolis, IN, USA). The top layer of cell media and sucrose was thoroughly removed and the pellet was soaked in sterile PBS for 1 h on ice and resuspended. For sucrose gradient experiments, preparation of a linear sucrose gradient was described previously [34], 1 mL of the gradient was removed before the resuspended VLPs were layered onto the gradient and centrifuged with a Beckman Coulter SW 41 rotor at 11,000× *g* for 12 h at 4 °C. A total of 10 1-mL fractions were removed from top, diluted 3× with sterile PBS, and centrifuged at 12,000× *g* for 1 h at 4 °C on a bench-top centrifuge. Supernatants were completely removed and pellets were soaked in sterile PBS for 1 h on ice before being resuspended.

### 2.3. Antibodies and Immunoblotting

The anti-G protein monoclonal antibody (mAb) 3D3 was provided by Trellis Bioscience (Redwood City, CA, USA); the anti-F protein mAb motavizumab was provided by MedImmune (Gaithersburg, MD, USA); palivizumab was from Dr. Martin Moore’s laboratory; rabbit serum anti-M antibody was provided by Dr. Oomens (Oklahoma State University); and goat anti-RSV antibody was obtained from Millipore (Burlington, MA, USA). All anti-species fluorescence-conjugated secondary antibodies used in immunoblotting were obtained from LI-COR biosciences (Lincoln, NE, USA). All HRP-conjugated secondary antibodies used in enzyme-linked immunosorbent assays (EIAs) were obtained from Jackson ImmunoResearch (West Grove, PA, USA). For immunoblotting experiments, VLP samples were mixed with 2× Laemmli sample buffer (Bio-Rad, Hercules, CA, USA) and boiled at 95 °C for 5 min. Samples were run on SDS-PAGE, transferred to a nitrocellulose membrane, blocked for 30 min in blocking buffer (5% dry milk in TTBS, 0.1% Tween-20 in tris-buffered saline). The primary antibody was added and incubated for overnight at 4 °C, membrane was washed 3× in TTBS, secondary antibody added and incubated for 30 min, and the membrane washed 3 times in TTBS. Signals were visualized by Odyssey CLX imaging system (LI-COR).

### 2.4. F, Ga, and Gb Antibody EIAs

The secreted form of F, Ga, or Gb protein antigens was produced from stably-transfected 293F cells in serum-free media and coated onto a 96-well microtiter plate in coating buffer (15 mM Na_2_CO_3_, 35 mM NaHCO_3_, 3 mM NaN_3_, pH 9.4–9.6). The plates were incubated in 2% nonfat dry milk dissolved in PBS blocking solution for 2 h at 37 °C, washed with PBS-T (PBS + 0.15% Tween-20), and serum specimens at 1:200 dilution added to the wells, incubated for 1 h at 37 °C, the plates washed with PBS-T, and goat anti mouse IgG-HRP (1:5000) added and incubated for 1 h at 37 °C. Color was developed with *o*-Phenylenediamine dihydrochloride (OPD) (Sigma-Aldrich, St. Louis, MO, USA) substrate and the reaction stopped after 30 min at RT with 4N H_2_SO_4_. The absorbance was measured at an optical density (OD) of 490 nm and the geometric mean of the OD_490_ was calculated from the triplicate wells.

### 2.5. RSV Neutralizing Antibody Assay

Heat inactivated sera were serially two-fold diluted starting with a 1:10 dilution in minimum essential media (MEM) (Gibco, Thermo Fisher Scientific, Waltham, MA, USA) containing 0.5% fetal bovine serum (FBS) (R&D Systems, Minneapolis, MN, USA), incubated with RSV/A2 (100 TCID_50_) for 1 h at RT, and inoculated in triplicates onto non-confluent HEp-2 monolayers in 96-well plates for 1 h at 37 °C in a 5% CO_2_ incubator. MEM containing 5% FBS was added to all the wells and cells were incubated for 3 days at 37 °C in a 5% CO_2_ incubator. The plate was washed with PBS and fixed with 4% paraformaldehyde. The plate was washed with PBS and incubated in PBS containing 0.3 M glycine for 30 min at RT followed by incubation in blocking solution (1% gelatin + 1% casein + 1% dry milk in PBS) for overnight at 4 °C. The plate was then incubated with goat anti RSV antibody (1:5000) followed with donkey anti-goat IgG-HRP secondary antibody (1:5000). Both primary and secondary antibodies were diluted in blocking buffer supplemented with 0.15% Tween-20. Color was developed with OPD substrate and neutralization defined as a ≥ 50% reduction in OD value. The titer was estimated using the Reed and Muench method [35]. The geometric means ± SEM for all animals in a group at any given time were calculated.

### 2.6. VLP Negative-Stain Transmission Electron Microscopy

Conventional negative-stain transmission electron microscopy (TEM) and immunolabeling was performed, as described previously [36]. Briefly, 4 μL of diluted samples was applied onto glow-discharged EM grids (FCF300-Cu; Electron Microscopy Sciences, Hatfield, PA, USA), washed with distilled pure water, stained in droplets of 0.75% uranyl formate (UF, pH 4~5) or 1% phosphotungstic acid (PTA, pH 6~7) for 1-min. The staining was carried out on an ice block. The grids were then blotted from the backside and air-dried inside a petri dish for at least 30-min at room temperature to minimize the negative-stain artifacts of flattening and stacking [37]. The negative-stain grids were imaged in low-dose mode (50 e^−^/Å), using a FEI Tecnai 12 transmission electron microscope (Thermo Fisher Scientific, previously FEI, Hillsboro, OR, USA) at 120 kV, images were acquired on a 4k × 4k Gatan OneView Camera (Pleasanton, CA, USA).

### 2.7. Immunogold Labeling Transmission Electron Microscopy

VLPs were subjected to immunogold labeling. The procedures were adapted from a previous published method [38]. A total of 4 μL of diluted samples was incubated on glow-discharged EM grids (FCF300-Ni; Electron Microscopy Sciences, Hatfield, PA, USA) for 5 min at room temperature. The grids were incubated on droplets of 20 mM glycine for 10-min, followed by a 30-min blocking in 5% bovine serum albumin in PBS without calcium chloride, without magnesium chloride (DPBS, [−] Ca, [−] Mg). The grids were floated onto 30 μL droplets of palivizumab (1:50) or Motavizumab (40 μg/mL) in 5% BSA in DPBS ([−] Ca, [−] Mg) overnight at 4 °C. After three washes with 1% BSA in DPBS ([−] Ca, [−] Mg), 5-min per wash, the grids were placed on 30 μL of droplets of 6-nm gold beads conjugated with goat anti-human polyclonal IgG antibody (Electron Microscopy Sciences, Hatfield, PA) at 1:100 dilution in 5% BSA in DPBS ([−] Ca, [−] Mg), for 1-h at room temperature. Then the grids were washed three times with 1% BSA in DPBS ([−] Ca, [−] Mg), and stained with 1% PTA (pH 6~7), as described above. The negative-stain grids were imaged in low-dose mode (50 e^−^/Å), using a FEI Tecnai 12 transmission electron microscope (Thermo Fisher Scientific, previously FEI, Hillsboro, OR) at 120 kV, images were acquired on a 4k × 4k Gatan OneView Camera (Pleasanton, CA, USA).

### 2.8. VLP Morphology Characterization

Only purified particles with a clear presence of surface glycoproteins were quantified, measured, and analyzed. Particle 2D-projection surface area was measured in IMOD using the *imodinfo* command by making close-contour objects [39]. The circular equivalent diameter X_A_, defined as the diameter of a circle with the same area as the particle, was calculated per equation X_A_ = $\sqrt[{2}]{\frac{4A}{\pi }}$, where A is the surface area of the VLPs, expressed in units of nm [40]. Similarly, the maximum Feret’s diameter, defined as the furthest distance between any two parallel tangents on the particle, was measured in IMOD, expressed in units of nm [40]. The distribution of the particle measurements was displayed in a histogram graph generated in Prism software (GraphPad Software, San Diego, CA, USA).

### 2.9. Virus

A recombinant virus of the RSV A2 backbone expressing the F protein from L19 virus (r19F) [41] was chosen as the challenge virus since it induces airway disease that parallels RSV infection in humans but is not seen with RSV A2. Stock virus was prepared by inoculating sub-confluent cultured HEp-2 cells with the recombinant RSV A2 at a multiplicity of infection (MOI) of 0.01 for 2 h at 37 °C in 5% CO_2_ incubator using 0.5% FBS-containing MEM. 5% FBS MEM was added and cells were incubated at 37 °C with 5% CO_2_ for three days. Cells were frozen and thawed twice at −80 °C and 4 °C, respectively, and centrifuged at 2000 rpm for 15 min at 4 °C to remove cellular debris. The supernatant was layered onto 20% sucrose and centrifuged at 12,200× *g* for 2 h at 4 °C. The pellet was resuspended in serum free MEM, divided into aliquots, and snap frozen in liquid nitrogen. The aliquots were stored at −80 °C until used. The infectivity titer of the inoculum was determined by serial 10-fold dilutions in sub-confluent HEp-2 cell cultures for three days and virus replication detected by EIA for RSV antigens with goat-anti RSV antibody. Titer was estimated from wells with absorbance > 3 standard deviations above the mean absorbance for wells without virus by the Reed and Muench method.

### 2.10. Animal Study

Four- to six-week old female BALB/c mice were purchased from Charles River Lab (Wilmington, MA, USA) and housed at Emory’s Department of Pediatrics animal facility under food ad libitum in micro-isolator cages with auto sterilized water. All animal handling and procedures were carried out according to protocols approved by Emory University (Atlanta, GA, USA) Institutional Animals Care and Use Committee (IACUC), (PROTO201700401, approved on 06/12/2018). For challenge studies, mice were intranasally infected with 10^6^ TCID_50_ RSV r19F A2, 40 μL in volume.

### 2.11. Real-Time PCR

Total RNA was extracted and purified from lung homogenates using Qiagen RNeasy kit (Qiagen, Hilden, Germany). RNA was reverse transcribed into cDNA using iScript™ cDNA synthesis kit (Bio-Rad) following the manufacturer’s instruction. Quantitative PCR was carried out on a 7500 Fast Real-time PCR system (Applied Biosystems, Foster City, CA, USA) using Power SYBR Green PCR master mix (Applied Biosystems). C_T_ values were normalized using control β-actin C_T_ values from the same samples. RSV matrix M gene primers and amplification cycles were described previously [42]. Other primer pairs used were: β-actin, forward 5′-CAC CAA CTG GGA CGA CAT-3′, reverse 5′-ACA GCC TGG ATA GCA ACG-3′. mRNA levels were expressed as the geometric mean ± SEM for all animals within a group.

### 2.12. Pulmonary Histopathology

Lungs were isolated and fixed in 10% neutral buffered formalin for 24 h. The lungs were then embedded in paraffin, sectioned, and stained with Periodic acid-Schiff (PAS). The slides were scanned on Hamamatsu Nanozoomer (Hamamatsu Corporation, Bridgewater, NJ, USA), analyzed by Aperio ImageScope software version 12.4.0.5043 (Leica Systems Inc., Buffalo Grove, IL, USA), and scored blinded to treatment on a 0–4 scale. They were subsequently converted to a 0–100% histopathology scale.

### 2.13. Statistical Analysis

Unless otherwise indicated, different groups were compared by Wilcoxon rank sum test or Wilcoxon matched pairs test. A *p*-value of <0.05 was considered statistically significant. Data are shown as means and standard errors of the mean (SEM).

## 3. Results

### 3.1. Generation of G and F Virus-Like Particles (VLPs) on RSV M and P or M and M2-1 Platforms

**Figure 1:**
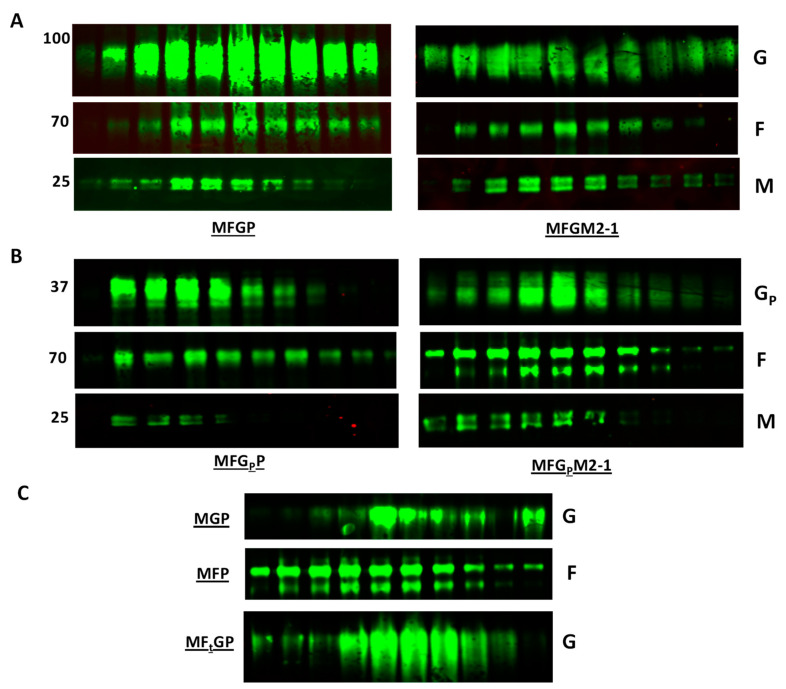
Generation and expression of F and G on RSV VLPs. 293F cell line expressing RSV gene M, F (or F_t_), G (or G_P_), and P or M2-1 were induced for 72 h in 2 μg/mL doxycycline. Cells were harvested and low-speed centrifugation performed to separate cells and VLPs-containing supernatant. VLPs were filtered through 0.45 μm filter to clear cell debris, layered on top of a 20% sucrose cushion and subjected to centrifugation at 12,200× *g* for 2 h, 4 °C. VLP pellets were resuspended in sterile PBS and subjected to centrifugation through a 20–60% sucrose gradient at 11,000× *g* for 12 h, 4 °C. A total of 10 fractions were collected and analyzed by immunoblotting using 3D3 (human anti-G antibody), motavizumab (human anti-F antibody), and rabbit serum anti-M antibody. (**A**) VLPs MFGP and MFGM2-1. Note that high levels G, F, and M are detected in similar fractions, (**B**) VLPs MFG_P_P and MFG_P_M2-1. G_P_, truncated G: aa 1-86+155-206. Note that high levels G, F, and M are detected in similar fractions, (**C**) MGP, MFP, and MF_t_GP VLPs. F_t_, truncated F: aa 496–574. Note that the expression level of G is less without F (MGP) than when F_t_ is co-expressed with G (MF_t_GP).

RSV VLPs were generated by sequentially transfecting 293F cells with codon-optimized DNA plasmids containing RSV genes. The order of transfection is listed in Table 1. Western blot studies suggested that we successfully developed VLPs with RSV M plus P or M plus M2-1 protein platforms with F or F and G (Figure 1A) but only to very low levels with G alone, a condition not observed with F in VLPs expressing only F (Figure 1C), i.e., detection of F or F and G in the supernatant and not the cell pellet of the induced 293F cells. VLPs are released from cells. Additionally, F or F and G were detected by Western blot in the pellet after centrifugation through a sucrose cushion indicating the proteins were incorporated into particles and not in solution in the media. Finally, F and M, or F, G, and M were detected by Western blot in the same fractions after sucrose gradient purification indicating presence of M in VLPs containing F and F and G (Figure 1A).

Given the importance of G’s central conserved domain in disease pathogenesis, we chose to develop a VLP that would focus the G immune response to this region. To this end, co-expressed F and G peptides with the intracellular, transmembrane, and first 20 amino acids of the extracellular domains (amino acids 1–86) plus amino acids 155–206 representing the central conserved domain (G_P_). We successfully generated these VLPs with either the M+P or M+M2-1 platform as indicated by Western blot studies of sucrose gradient purified VLPs (Figure 1B).

The fact that G alone was inefficiently but with F was efficiently incorporated into the VLPs suggested that F facilitated generation of G VLPs. The importance of F in RSV VLP formation has been noted in an earlier study showing that the carboxyl terminal portion of F is required for VLP formation [31]. We hypothesized that the intracellular and transmembrane F would be sufficient to form G VLPs and generated a truncated F with its carboxyl terminus + transmembrane + first 26 amino acids of the extracellular domain (F_t_) to co-express with full-length G. Our data with the M + P VLP platform show that with F_t_, G is abundantly expressed in the VLPs (Figure 1C).

### 3.2. Electron Microscopy Charateristics of G and F on VLPs

**Figure 2:**
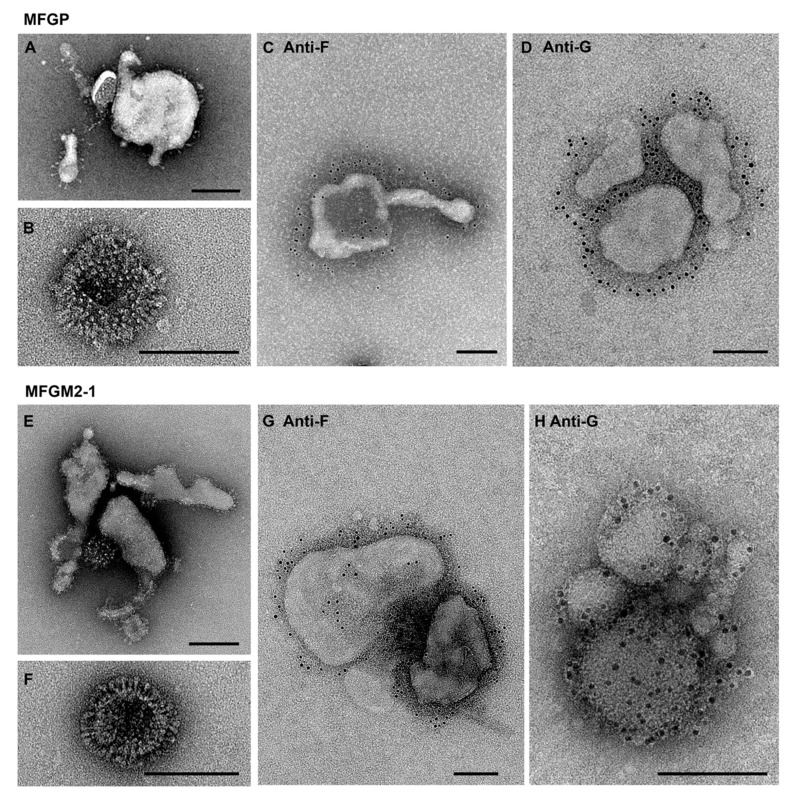
Electron microscopy studies show glycoproteins on the surface of RSV VLPs. Negative stain electron microscopy images showed that both MFGP and MFGM2-1 VLPs were of various shapes and sizes, displaying clear spikey glycoprotein densities on the surface (**A**,**B**,**E**,**F**). Immunogold labeling confirmed the presence of glycoprotein F with Palivizumab (**C**,**G**), and glycoprotein G with 3D3 (**D**,**H**) on the surface of MFGP and MFGM2-1, respectively. All scale bars are 100 nm.

To confirm generation of F and G VLPs, negative-stain transmission electron microscopy (NS-TEM) was performed on both MFGP and MFGM2-1 particles. NS-TEM examination showed that the VLPs with both constructs have the expected protein-like electron densities corresponding to surface glycoprotein F and G on their surface (Figure 2A,B,E,F). We confirmed the presence of the two surface glycoproteins on the VLP surface with immunogold labeling as illustrated in Figure 2.

**Figure 3:**
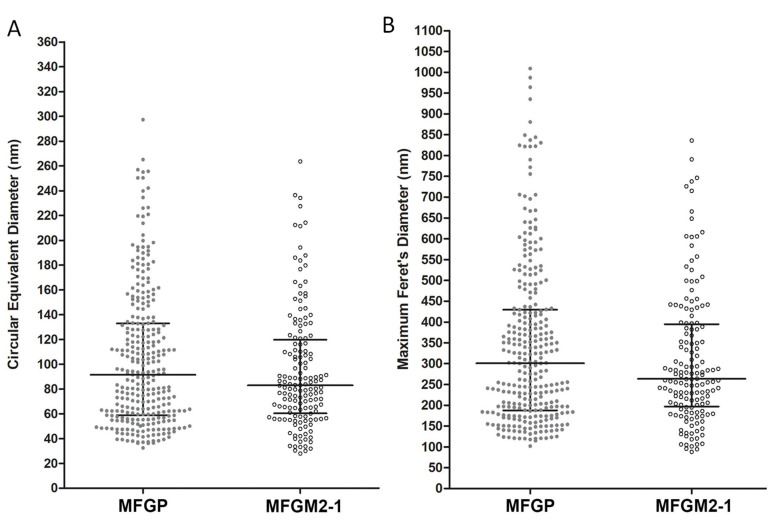
Size distribution of MFGP and MFGM2-1 VLPs. Morphological analysis was performed on MGFP and MFGM2-1. Circular equivalent diameter (**A**) and maximum Feret’s diameter (**B**) were measured and analyzed from negative stain electron microscopy images of MFGP (*n* = 295) and MFGM2-1 (*n* = 151). Median and interquartile ranges are shown in the graph. The median of the circular equivalent diameter is 91.6 nm for MFGP, and 83.05 nm for MFGM2-1. The median of the maximum Feret’s diameter is 300.8 nm for MFGP, and 263.4 nm for MFGM2-1.

To quantify size and morphologic characteristics, purified VLPs were imaged using 2D NS-TEM (Figure 3). VLP surface area and maximum Feret’s diameter (MFD) showed large variation in size for MFGP VLPs. with circular equivalent diameter (CED) ranging from 32 to 298 nm (Figure 3A). CED is defined as the diameter of a circle with the same area as the particle. Approximately, 80% of MFGP VLPs (*n* = 295) are 50~200 nm in CED and 100~500 nm in MFD, with a median of 91.6 nm and 300.8 nm, respectively (Figure 3). The other VLP platform MFGM2-1 showed a similar variation in size and shape, with CED from 28 to 263 nm and MFD from 88 to 836 nm (Figure 3). Almost 90% of MFGM2-1 (*n* = 151) fell in the range of 32–160 nm in CED (the median = 83.05 nm) and 88–490 nm in MFD (the median = 263.4 nm). Despite the artifacts introduced by 2D NS-TEM such as stacking and flattening, it is clear that MFGP and MFGM2-1 VLPs are highly variable in shape and size.

### 3.3. VLPs Are Immunogenic

**Figure 4:**
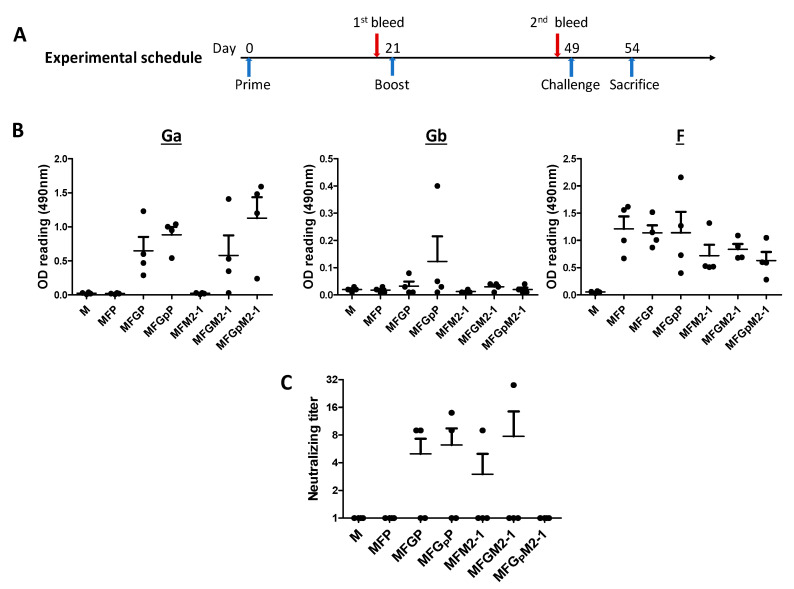
VLP-immunized mice generate serum antibodies against G and/or F. (**A**) Schematic schedule of animal experiments. (**B**) Sera from immunized animals (diluted 1:200) were tested by ELISA for Ga, Gb, or F protein-binding antibodies. After blocking, plates were incubated with goat anti mouse IgG-HRP secondary antibody. OPD substrate was used to develop reaction and absorbance at 490 nm was read. Note the VLPs efficiently induced antibodies against Ga and F but not Gb. (**C**) Sera from immunized animals were heat inactivated at 56 °C for 30 min followed by two-fold serial dilution in triplicate. The dilutions were incubated with 100 TCID_50_ of RSV A2 virus for 1h at RT. The mixtures were then transferred to monolayer HEp-2 cell and incubated for 1 h at 37 °C in 5% CO_2_. 5% FBS + MEM media was added to the cells followed by incubation for 72 h at 37 °C in 5% CO_2_. Cells were fixed and ELISA was performed using goat anti-RSV antibody and HPR-conjugated donkey anti-goat secondary antibody. Reaction was developed by OPD and absorbance read at 490 nm. Neutralizing titers were calculated using Reed–Muench method. Note low levels of neutralizing antibodies were induced by the VLPs.

To determine the immunogenicity of the VLPs, we immunized BALB/c mice (*n* = 4) with the VLPs as detailed in Table 2. All immunized animals were challenged with 10^6^ TCID_50_ of RSV r19F at four weeks after the second, booster, immunization (Figure 4A). Blood specimens were collected before challenge and tested for F, a group A G (Ga), and a group B G (Gb) protein binding antibodies by EIA and neutralizing antibodies by a micro-neutralization assay. All immunized animals except for those immunized by control M VLPs developed antibodies against F and Ga. Only one animal produced antibodies against Gb antigen (Figure 4B). Additionally, the short G peptide (G_P_) seemed to be more efficient at inducing anti-Ga antibody than its full-length counterpart (*p* > 0.05), indicating that the central conserved domain of G is an effective immunogen. Furthermore, VLP antigens that contained P seemed to induce antibodies against F better than those that contained M2-1 but this difference was not significant (Figure 4B). In this study, the VLPs induced low levels of neutralizing antibodies. As shown, sera from animals in groups immunized with MFP and MFM2-1G_P_ did not possess neutralizing activity but sera from other groups had one or two animals with some low titer neutralizing activity (Figure 4C). This difference was not significant.

### 3.4. VLP Immunization Reduces Lung Viral Titers

**Figure 5:**
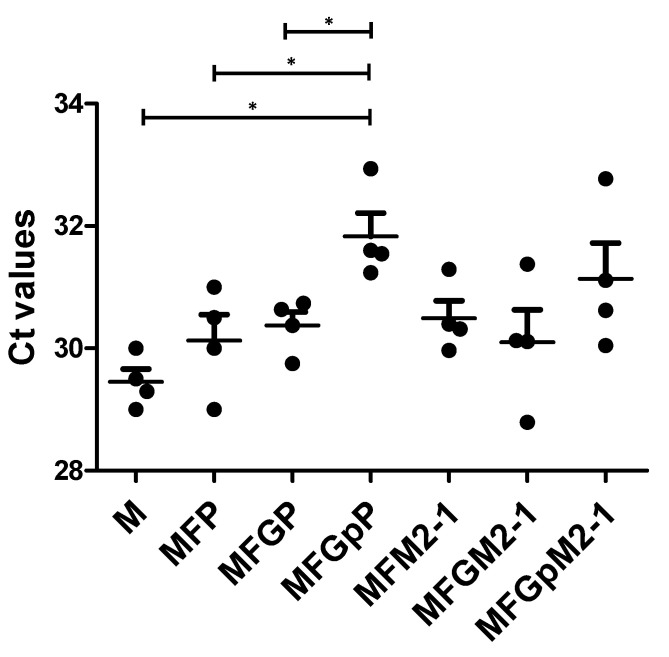
Immunized animals have significantly less lung viral titer. Lungs from immunized animals were homogenized as described in materials and methods. Aliquots stored at −80 °C were thawed and total RNA was extracted from lung. RNAs were then reverse transcribed into cDNAs. These were used as templates in RT-PCR using CYBR green and a pair of RSV matrix protein M specific primers as described. In parallel, similar reactions were performed using a pair of β-actin specific primers as controls. Results were expressed as relative amount of RSV M compared to β-actin. * *p* < 0.05. Note the VLPs with Gp appeared most effective at reducing virus replication as indicated by PCR C_T_ values.

Next, we investigated the ability of the VLPs to prevent virus replication after challenge. We purified total RNA from lung homogenates and performed RT-PCR using RSV M matrix protein primers and β-actin as control. Figure 5 shows that the relative cycle threshold (C_T_) values were significantly higher indicating less virus replication in animal groups that had P protein as part of the antigen VLPs, i.e., MFP, MFGP, and MFG_P_P compared to the control antigen (M only VLPs). A higher value of C_T_ correlates with low copy of the gene being evaluated. Additionally, there were significant differences between MFG_P_P and MFP or MFGP (Figure 5), indicating that anti-G antibodies participate in viral clearance in the lungs and that G_P_ was more effective in facilitating virus clearance than full length G. Moreover, VLP antigens that contained M2-1 protein, i.e., MFM2-1, MFGM2-1, and MFG_P_M2-1 had C_T_ values similar to those from the comparable construct on the M+P platform, but not significantly higher than control C_T_ values (Figure 5).

### 3.5. VLP Immunization Reduces Lung Inflammation

**Figure 6:**
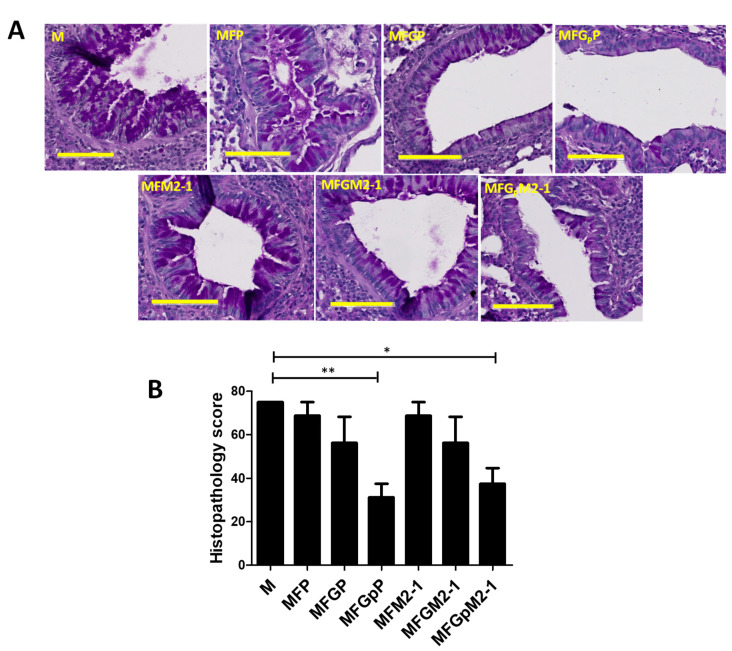
Immunized animals show a significant reduction in lung inflammation. Female BALB/c mice (4–6 weeks) were divided in 7 groups (*n* = 4), immunized, and challenged as summarized in Table 2. Lungs were collected, fixed, and stained with periodic-acid Schiff (PAS) staining as described in the materials and methods. The slides were analyzed by Aperio ImageScope software and scored blindly on a 0–4 scale and subsequently converted to a 0–100% histopathology, Bar = 100 µm scale. (**A**) Representative images from corresponding groups. (**B**) Quantitative data converted from histopathology scale. * *p* < 0.05, ** *p* < 0.01. Note the VLPs with Gp appeared most effective at reducing lung mucin levels.

One of the manifestations of RSV infection with r19F virus is the overproduction of mucin [41], therefore, we examined pulmonary inflammation in challenged animals by Periodic acid-Schiff staining (PAS). The stained slides were analyzed by Aperio ImageScope software (Leica, Germany) and scored blindly using 0–4 severity scale and then converted to 0–100 histopathology scale. Here, positive PAS staining was observed in the lungs of all animals, indicating the presence of mucin. However, animals immunized with VLPs expressing F and/or G showed decreased staining compared to animals immunized with the control VLPs expressing only M. (Figure 6A,B). Only MFG_P_P- and MFG_P_M2-1-immunized animals had a significant reduction in PAS staining when compared to control animals (Figure 6A,B). These data show that F plus Gp VLPs were the most effective at reducing both lung virus replication and mucin production.

## 4. Discussion

We developed two RSV-based VLP platforms M+P and M+M2-1 expressing F and G, F and G peptide, or Ft and G. The EM studies show surface projects and variability in size and shape. The virus is also pleomorphic in size and shape [43]. Important to structural studies and vaccine development, these two platforms support the generation of VLPs with different F and G constructs. For structural studies, generation of G and truncated F (F_t_) VLPs makes it possible to study G without most of the F ectodomain. These VLPs provide a tool to study structure, function, and immunogenicity of G with minimal influence from extracellular F. Studies of G with Ft also provide the framework to identify changes associated with the addition of F. From a vaccine perspective, we wanted to be able to focus immune responses to certain regions of G. Given the importance of the central conserved domain of G to disease pathogenesis [17,22,23,42,44], we chose to focus an immune response to this region. We successfully generated VLPs with a truncated G that expressed a peptide from the central conserved domain of G on its surface and immunization with this VLP compared with one with full-length G reduced lung virus titer and lung inflammation more effectively, though not significantly. This improved protection is not surprising since VLPs with Gp induced higher titer of anti-G antibodies which should be against central conserved domain of G. Antibodies to this region of G have been shown to both decrease virus replication and lung inflammation in mice [16,17,19,21].

The RSV M+P VLP platform was described earlier [31] but the RSV M+M2-1 VLP platform was not. The M2-1 protein has transcription anti-termination activity and directly interacts with M, providing a link to the RNA-containing nucleocapsid [26,43,45,46,47]. The M2-1 protein’s interaction with M provides a basis for considering ways it might support M+M2-1 VLP formation, e.g., by stabilizing M on the VLP surface. How P facilitates VLP formation has not been determined. P does have a crucial role in RSV polymerase activity by interacting with both nucleocapsid (N) and polymerase (L) proteins [48,49,50] and also cooperates with M2-1 [51]. However, these roles do not suggest how it might support VLP formation when co-expressed with M.

Negative stain EM studies revealed that the two VLP platforms expressed the surface glycoproteins F and G equally well with no structural differences observed. Similarly, our mouse studies show that both VLPs were immunogenic and induced serum antibodies against both F and G proteins to similar levels and were effective in decreasing lung virus and inflammation in RSV challenged mice. Interestingly, the VLPs with Gp compared to VLPs with full length G induced higher antibody titers and were more effective in decreasing lung virus and lung inflammation. The M2-1 protein’s ability to induce short term protective T cell immunity in mice [30,52,53] might theoretically be advantageous in a vaccine. Whether this is an advantage for M+M2-1 versus M+P VLPs requires further study. We did not measure T cell responses in this study. Of note, the G protein sequences used here are from a group A strain (A2) and these G sequences efficiently induced antibodies against a group A but not a group B G protein. This suggests that G used in an RSV vaccine will likely need to have both group A and B sequences. Much of CCD-G is conserved within but not between groups. We also tested serum neutralizing antibody titers and observed that immunized animals developed low levels of neutralizing antibodies. VLPs with a pre-fusion stabilized F protein might be more effective at inducing neutralizing antibodies [54,55,56]. Further study is needed to determine the differences between the two platforms that may affect structure, function, immunogenicity, or other features of the VLPs, and to improve immunogenicity of the VLPs for a vaccine.

In summary, we successfully developed VLPs expressing F and/or G with two RSV platforms, co-expression of the M+P proteins or M+M2-1 proteins. With both platforms we were able to modify F and/or G to focus specific structural and antigenic features of the proteins. These platforms provide a flexible way to study the structure and function of these two proteins and to develop RSV subunit vaccines.

## Figures and Tables

**Table 1:** Generation of VLPs. 293F/6TR cells were sequentially and permanently transfected with plasmid DNA coding for RSV proteins as shown in table. G_P_: G protein peptide (aa 1-86+155-206).

VLPs	Order of Transfection			
	1st	2nd	3rd	4th
M	M			
MFP	M	F	P	
MFPG	M	F	P	G
MFPG_P_	M	F	P	G_P_
MFM2-1	M	F	M2-1	
MFM2-1G	M	F	M2-1	G
MFM2-1G_P_	M	F	M2-1	G_P_

**Table 2:** Immunization schedule. Mice were divided into seven groups and immunized as shown above and in Figure 4A. Other groups: MFP, MFGP, MFG_P_P, MFM2-1, MFGM2-1, or MFG_P_M2-1. IM, intramuscular.

Group	*n*	Dose (Per Mouse)	Immunization Days	Route	RSV Challenge TCID_50_/Mouse	Days of Harvest
M	4	50 μg VLPs	0, 21	IM	10^6^ at day 49	54
Others	“	“	“	“	“	“
